# Notes on Preparations for the Sick

**Published:** 1913-03

**Authors:** 


### Notes on Preparations for the Sick.

**Glycero-Phosphates: Rubelix.**—H. E. Matthews & Co., Clifton.—This new glycero-phosphate compound is a palatable liqueur, free from sugar, but having a similar composition to the usual glycero-phosphate syrups. Each ounce contains one-sixteenth of a grain of strychnine, and half a grain of quinine. The dose is a teaspoonful, increased to a table-spoonful, to be taken alone or with water, as desired. It is a clear red fluid, which does not precipitate on dilution. It has a slight chalybeate and bitter taste, and its composition should endow it with the usual tonic properties of this class of compound.

**"Tabloid" Thyroid Gland, gr. $\frac{1}{10}$ and gr. $\frac{1}{4}$.**—Burroughs, Wellcome & Co., London.—In thyroid medication the dose of thyroid gland has usually been regarded as 3 to 10 grains. Recently, however, certain authorities have stated that as the result of extensive clinical trials they have found doses of gr. $\frac{1}{10}$ to gr. 1 equally satisfactory. To provide for this new system of dosage two additional strengths—gr. $\frac{1}{10}$ and gr. $\frac{1}{4}$— of "Tabloid" Thyroid Gland have been issued. A large number of the most brilliant results recorded in the literature of thyroid therapy have been obtained with "Tabloid" Thyroid Gland, which has been proved to be an exceedingly active and reliable preparation. It is standardised by chemical means, so as to ensure that the desiccated gland substance, of which each product represents a definite amount, contains not less than 0.2 per cent. of iodine in organic combination.

The strengths in which "Tabloid" Thyroid Gland is issued now include gr. $\frac{1}{10}$, gr. $\frac{1}{4}$, gr. $\frac{1}{2}$, gr. 1, gr. $1\frac{1}{2}$, gr. $2\frac{1}{2}$, gr. 5, 0.05 gramme, 0.1 gramme, and 0.3 gramme.

---

**Tuberculin Bouillon Filtrate, Human (T.O.A.). Tuberculin Bouillon Filtrate, Bovine (P.T.O.).**—Burroughs, Wellcome & Co., London.—Two new preparations have been added to the list of "Wellcome" Brand Tuberculins. Each is issued in hermetically-sealed phials of 1 c.c., both undiluted, and in dilutions representing 0.0001 c.c., 0.001 c.c., 0.01 c.c., and 0.1 c.c of T.O.A. or P.T.O. The undiluted preparations are for the use of practitioners who prefer to make their own dilutions. The bouillon filtrates T.O.A. and P.T.O. were the earliest tuberculins prepared, though not the first introduced into therapeutics. Being the weakest of all the tuberculins, they are now employed by many workers to initiate a course of tuberculin treatment according to the intensive system of dosage, the use of which has of late become so widespread.

---

**Bolus Soap.**—Liermann, Actien-Gesellschaft für Anilin-Fabrication, Berlin.—This aseptic Bolus washing paste is intended for cleansing the hands before and after surgical procedures. It is a soft, paste-like substance, containing clay in the form of Bolus alba, which serves the part of a nail brush, and cleanses far more quickly. It is slightly scented, and is mounted in tubes ready for use. It is claimed that the Bolus method, applied for three minutes, leaves the hands practically free from germs, and the hands prepared by this method, and covered with rubber gloves, were found to be free from germs three hours later.

